# Intragastric Botulinum Toxin Injection and Botulism: An Alarm for Clinicians

**DOI:** 10.1155/2024/8183127

**Published:** 2024-04-12

**Authors:** Mojtaba Mojtahedzadeh, Farhad Najmeddin, Elham Pourheidar, Atabak Najafi, Reza Bahman, Ehsan Yousefi-Mazhin, Hossein Karballaei-Mirzahosseini, Rezvan Hassanpour

**Affiliations:** ^1^Department of Clinical Pharmacy, Faculty of Pharmacy, Tehran University of Medical Sciences, Tehran, Iran; ^2^Department of Intensive Care Unit, Rasoul Akram Hospital, Iran University of Medical Sciences, Tehran, Iran; ^3^Department of Anesthesiology and Critical Care, Sina Hospital, Tehran University of Medical Sciences, Tehran, Iran; ^4^Faculty of Medicine, Tehran University of Medical Sciences, Tehran, Iran

## Abstract

Clostridium botulinum produces the most potent bacterial toxin, botulinum toxin A (BTXA), which has various therapeutic and cosmetic indications. Intragastric BTXA injection is a new obesity treatment method that was argued to be safe due to the inactivation of BTXA through the liver or metabolization within the gastric wall. However, a 36-year-old woman was admitted to the intensive care unit (ICU) due to developing botulism as a result of an intragastric injection of BTXA. The diplopia, headaches, ptosis, decreased muscle force, and respiratory distress two days after injection were her first chief complaints, and also, she experienced significant dysphagia, hoarse voice, thick tongue, constipation, hyposmia, and hypogeusia after two weeks. This case report highlights the necessity for physicians to have sufficient information about this method and consider possible life-threatening adverse effects including botulism.

## 1. Introduction

Botulinum toxin A (BTXA), produced by Clostridium botulinum, is the most potent bacterial toxin with therapeutic and cosmetic indications, such as strabismus, blepharospasm, hemi facial spasm, cervical dystonia, pediatric cerebral palsy-related spasticity, hyperhidrosis, and bladder dysfunction [1]. Additionally, this toxin can be used for certain digestive diseases including achalasia and chronic anal fissure [2]. Endoscopic intragastric BTX injection is a novel treatment for morbid obesity due to gastric emptying delay and early satiety [3].

Based on the benefit-risk paradigm for obesity devices-therapies approved by the US Food and Drug Administration (FDA), the risk of this method was categorized as class I (the lowest) [4], but there have been reports of developing iatrogenic botulism around the world [5, 6]. For example, 34 cases in four European countries, with sources of Turkish hospitals, developed symptoms with a median of three days after intragastric BTX injection that led to the hospitalization of 26 cases [6].

In this article, we will describe a case, in which botulism developed after receiving intragastric of BTX, and we will outline her management in our medical setting.

## 2. Case Presentation

A 36-year-old woman was admitted to the intensive care unit (ICU) of Sina Hospital affiliated to Teheran University Medical Sciences (TUMS), two weeks after receiving an intragastric injection of BTXA. She complained of diplopia, headaches, ptosis, decreased muscle force, and respiratory distress two days after injection and additionally experienced significant dysphagia, hoarse voice, thick tongue, constipation, hyposmia, and hypogeusia after two weeks.

At the time of admission, the patient was conscious, oriented, and cooperative. Her hemodynamic parameters were stable and intubation was not required.

Given the clinical presentation and history of BTXA injection, one vial (10 ml) of trivalent botulinum antitoxin (BAT) was diluted in 100 ml of normal saline and was infused 30 ml/hour (over 3 hours), alongside supportive care, to manage the patient's botulism syndrome.

After 24 hours of BAT injection, the patient's symptoms including dysphagia, hypogeusia, hyposmia, constipation, and hoarse voice showed significant improvement and other symptoms gradually regressed over one week. She was discharged from the ICU after 4 days.

## 3. Discussion

The syndrome of botulism occurs in several types including infant, foodborne, wound, inhalation, and iatrogenic botulism [7]. Iatrogenic botulism, a rare type, has been reported in patients who received BTXA for cosmetic or medical purposes [8–10].

Intragastric BTXA injection is a new obesity treatment method introduced in 2003, and a dose between 100 and 500 units diluted of BTXA was injected into the gastric antrum [3, 11]. It was initially considered safe due to the inactivation of BTXA through the liver or metabolization within the gastric wall [12], and only gastric reflux and hemorrhagic gastritis with gastric necrosis have been reported [11]. However, reports of botulism cases emerged [5, 13, 14] such as the outbreak of iatrogenic botulism in four European countries [6].

The underlying factors contributing to toxicity are unknown, but injection frequency, cumulative dose, and tissue damage due to needle insertion are possible factors [12]. For instance, injection of 1000 to 2500 units of BTXA was reported on the outbreak of four European countries [6]. We lacked information about the dose, application, and commercial formulation of BTXA, so physicians should be aware that the correct process of commercial formulation selection, preparation, and accurate selection of injection sites and applying the recommended drug dosage are necessary for the optimal treatment outcomes and to decrease possible life-threatening complications such as iatrogenic botulism.

The only specific treatment for botulism is BAT which should be administered promptly, regardless of onset of illness, in the individuals with suspected botulism and progressive symptoms. BAT prevents progression to respiratory failure by binding to circulating neurotoxins and preventing their binding to the neuromuscular junction [15]. Various forms of BAT are available globally. The equine serum heptavalent BAT is FDA-approved to treat noninfant botulism and the recommended dose is one vial infused at a rate of o.5 ml/min up to 2 ml/min [15], and an additional dose of antitoxin may be considered if there is suspicion of ingesting a high amount of toxin. The commercial form of BAT in our country is equine serum trivalent (A, B, and E type), so we extrapolated from FDA-recommended dose and administrated one vial (10 ml) with an infusion rate of 30 ml/hour (over 3 hours). During the infusion, the patient did not experience any hypersensitivity reaction, and other adverse effects and symptoms including dysphagia, hypogeusia, hyposmia, constipation, and hoarse voice significantly improved after 24 hours, obviating the need for an additional dose.

## 4. Conclusion

This case report underscores the importance of physicians possessing sufficient knowledge about potential adverse effects, optimal dose, and correct preparation and injection of intragastric BTXA for the prevention of life-threatening adverse effects including botulism.

## Data Availability

The data that support the findings of this study are available upon reasonable request from the corresponding author (RH).
